# Stability of Superprotonic CsH_2_PO_4_ Hermetically Sealed in Different Environments

**DOI:** 10.3390/ma15144969

**Published:** 2022-07-17

**Authors:** Cristian E. Botez, Israel Martinez, Alex D. Price

**Affiliations:** 1 Department of Physics and Astronomy, University of Texas at San Antonio, 1 UTSA Circle, San Antonio, TX 78249, USA; 2 Department of Physics, University of Texas at El Paso, 500 W. University Avenue, El Paso, TX 79968, USA; imartinez6@utep.edu (I.M.); adprice.c137@gmail.com (A.D.P.)

**Keywords:** fuel cell electrolytes, proton conductivity, impedance spectroscopy

## Abstract

Using powder X-ray diffraction and AC impedance spectroscopy, we have found that the superprotonic CsH_2_PO_4_ (CDP) phase is stable at T = 250 °C when sealed in different volumes (15 mL and 50 mL) of dry air or inert gasses. Under these conditions, CDP’s proton conductivity stays constant at 2.5 × 10^−2^ S·cm^−^^1^ for at least 10 h. On the other hand, removing the gas from the chamber leads to a sharp, two-order-of-magnitude drop in the proton conductivity. Our data show no evidence of a self-generated water vapor atmosphere in the chamber, and the gas pressure at T = 250 °C is several orders of magnitude below the pressures previously used to stabilize CDP’s superprotonic phase. These results demonstrate that hermetically sealing CDP in small gas-filled volumes represents a new method to stabilize the superprotonic phase, which opens new paths for large-scale applications of phosphate-based solid acids as fuel cell electrolytes.

## 1. Introduction

Fuel cells that operate in the intermediate temperature range between 150 °C and 300 °C present several advantages, such as a low cost, efficient cooling, and a high tolerance to catalyst poisoning. Electrolyte candidates for such fuel cells include MH_n_XO_4_ solid acids (M = Cs^+^, Na^+^, Rb^+^, K^+^, X = S,P and n = 1,2), which are compounds that do not require hydration as they conduct protons and not hydronium ions. In addition, phosphate-based solid acids eliminate the risk of H_2_S catalyst poisoning posed by their sulfate-based counterparts [1,2]. Among the phosphate-based solid acids that can function as fuel cell electrolytes at intermediate temperatures, CsH_2_PO_4_ (CDP) has been the most investigated [3,4,5,6,7,8,9]. It was found that heating it above 234 °C leads to a superprotonic phase transition, where CDP’s proton conductivity undergoes a three-order-of-magnitude jump (to ~2.5 × 10^−2^ S·cm^−1^) concomitantly with a monoclinic to cubic structural modification [10,11]. Unfortunately, the superprotonic CDP phase is not stable and rapidly dehydrates in an open atmosphere according to the following reaction:

2CsH_2_PO_4_ → Cs_2_H_2_P_2_O_7_ + H_2_O (1)

The dehydration can be avoided by keeping CDP either under a saturated water vapor atmosphere or under high pressure (~1 GPa) [4,5,6], but these methods are not well-suited for large-scale fuel cell applications. Several alternative approaches, including the use of SiO_2_-based composites [12,13,14,15,16], mixed cation compounds [17,18,19], and nanoparticle with different surfactants [20] have been proposed and have led to the enhancement of CDP’s superprotonic phase conductivity and stability. Yet, its dehydration could not be completely inhibited in the absence of high pressures or a saturated water vapor atmosphere.

Recently, we found that CDP pellets exhibit a constant superprotonic conductivity for several hours when sealed in dry air at T = 260 °C [21], a finding that has already been confirmed, e.g., with CDP/TiO_2_ composites [22]. Neither high pressures (in the GPa range) nor high humidity were applied to the samples in these experiments, so the macroscopic physical conditions and microscopic chemical or structural modifications responsible for the observed superprotonic phase stability generated further research and discussion. It was recently proposed, for example, that CDP might actually partially dehydrate up until the released water builds up a saturated water vapor atmosphere within the chamber, which would limit further dehydration and yield the observed stable high conductivity at 260 °C [23]. Other possible scenarios involve the heating-induced gas pressure increase in the sealed chamber, and/or the type of gas in the sample environment. Obtaining further insight into the macroscopic parameters and microscopic mechanisms responsible for stabilizing CDP’s superprotonic phase is particularly important for both applied and fundamental reasons. Indeed, this has the potential to open new paths for large-scale applications of phosphate-based solid acids as intermediate-temperature fuel cell electrolytes and to reveal highly efficient proton conduction mechanisms that might carry over to other physical and biological systems such as polymers, oxide ceramics, intercalation compounds, and bio-membranes.

Here, we report results from X-ray diffraction and AC impedance spectroscopy measurements on CDP samples that are hermetically sealed in chambers of different volumes that are filled with dry air, inert gases, or kept under vacuum. We designed our experiments, including pellet preparation, chamber design, and time-resolved impedance spectroscopy and X-ray diffraction measurement, with the goal of testing whether the CDP stabilization observed in [21] could actually be due to a saturated water vapor atmosphere self-generated by the sample through its partial dehydration. Our data show that the cubic superprotonic CDP phase is stable in dry air volumes of 15 mL and 50 mL. In both cases we found superprotonic conductivity values above 2 × 10^−2^ S·cm^−1^, holding for at least 10 h at 250 °C. This result, corroborated with the use of mesh electrodes, and with the total absence of any trace evidence of caesium pyrophosphate, Cs_2_H_2_P_2_O_7_, represents strong evidence that no dehydration occurs in CDP samples sealed in dry air under the above-mentioned conditions. We also found that replacing the dry air with inert gasses (Ar, N) had little effect on the stability of the CDP superprotonic phase. However, totally removing the gas from the chamber, i.e., keeping CDP in a vacuum environment, has a dramatic effect leading to a rapid three-order-of-magnitude decrease in the sample’s proton conductivity, which is an even steeper drop than the one observed when CDP was kept in an open atmosphere. This indicates that the gas pressure does play an important role in stabilizing CDP’s superprotonic phase, but its magnitude at 250 °C in our experiments is four orders of magnitude less than the pressures previously used [6,11] to avoid CDP’s chemical decomposition above the superprotonic transition temperature T_sp_. Our findings demonstrate that hermetically sealing CDP in a small gas volume represents a new method to prevent its chemical decomposition above T_sp_ without the use of high humidity or high pressures in the GPa range.

## 2. Materials and Methods

CDP was synthesized by slow evaporation from Cs_2_CO_3_ (Acros Organics, Geel, Belgium, 99.9%) and H_3_PO_4_ (Alfa Aesar, 85% *w*/*w* aqueous solution) in a 1:2 mol ratio. The resulting crystals were mechanically ground and pressed into pellets of a 1.3 cm diameter × 0.25 cm thickness at 0.65 GPa. X-ray diffraction (XRD) data were collected on a PANalytical Empyrean system (Malvern Panalytical Inc., Westborough, MA, USA) using Cu-Kα radiation (λ = 1.5418 Å) that was equipped with an Anton Paar XRK 900 high-temperature chamber and a PIXcel3D detector. Proton conductivity was measured on CDP pellets by AC impedance spectroscopy using a Solartron 1260 impedance analyzer (AMETEK Inc., Berwyn, PA, USA) coupled with a ProboStat^®^ sample holder. The pellets were placed inside custom-made hermetically sealed chambers, gently pressed between two mesh electrodes, and a 100 mV oscillating potential (6 MHz to 1 Hz frequency range) was applied in a standard two-point and four-wire setup.

## 3. Results and Discussion

**Figure 1:**
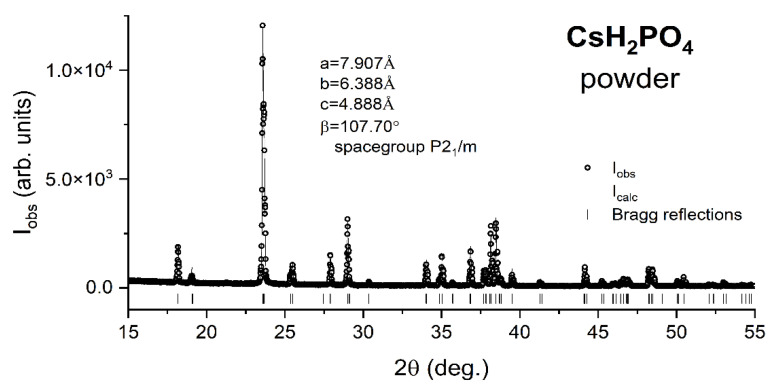
Le Bail (full profile) fit to X-ray diffraction data collected on the prepared CDP powders. The empty symbols show the observed intensity as a function of the detector angle 2θ and the solid line is the fit. The vertical bars mark the positions of the Bragg reflections from CDP’s room temperature monoclinic P2_1_/m unit cell of parameters a = 7.907 Å, b = 6.388 Å, c = 4.888 Å, and β = 107.70°.

The X-ray diffraction pattern I_obs_ vs. 2θ from the as prepared CDP powders is shown by the open symbols in Figure 1. The solid line represents a Le Bail fit that allows the lattice constants of CDP’s room temperature monoclinic unit cell (space group P2_1_/m) to be determined with high accuracy. In a Le Bail fit, the lattice parameters of the unit cell are refined together with the peak profile parameters and the “zero” (2θ_0_) of the pattern to obtain the best agreement between the observed and calculated intensities along the full profile of the pattern. In our case, the refinement converges upon the simultaneous variation of 9 parameters to yield lattice constants a = 7.907 Å, b = 6.388 Å, c = 4.888 Å, and β = 107.70°. In addition, we noticed that all peaks observed in the pattern correspond to the calculated Bragg reflections (marked by vertical bars), which demonstrates that no impurities are present in the as prepared powder.

**Figure 2:**
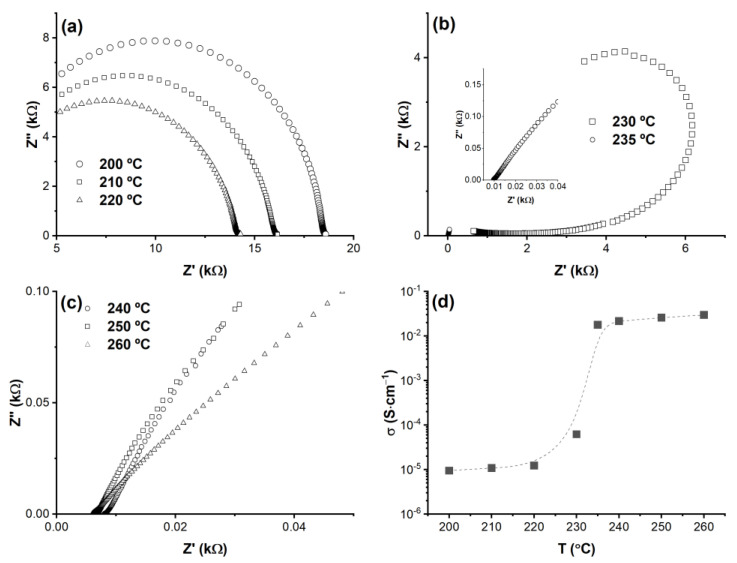
Nyquist plots measured on CDP pellets hermetically sealed in a 50 mL chamber filled with dry air (**a**) at temperatures below the superprotonic transition and (**b**) during the transition. The inset shows the data collected at high frequencies right above T_sp_ and (**c**) above the transition. (**d**) Temperature dependence of the proton conductivity within the 210–260 °C temperature range as obtained from the Nyquist plots (filled squares); the solid line is a guide to the eye. The data show the superprotonic jump at T_sp_ = 233 °C.

Figure 2a shows Nyquist plots measured below the CDP’s superprotonic transition temperature T_sp_ = 233 °C on CDP hermetically sealed in a 50 mL chamber in dry air at T = 200 °C (circles), T = 210 °C (squares), and T = 220 °C (triangles). The measured curves are semicircular segments corresponding to the slow proton diffusion below T_sp_, and, in each case, the proton conductivity was determined by the intersection of the semicircle with the in-phase impedance Z’ axis. Upon further heating, the Nyquist plot profiles changed significantly when nearing the transition. Indeed, the data recorded at T = 230 °C shown by the squares in Figure 2b gradually approach the Z’ axis, which becomes at a tangent to the data at a low Z’, corresponding to an already increased proton conductivity with respect to its T ≤ 220 °C counterparts. The data at T = 235 °C (circles) indicate that the CDP is already in its superprotonic state where the Nyquist plots denote a nearly straight segment, as more clearly shown in the inset to Figure 2b. Similar data, displayed in Figure 2c, were recorded at higher temperatures: T = 240 °C (circles), 250 °C (squares), and 260 °C (triangles) The segments shown here correspond to a 10 Hz to 1 × 10^5^ Hz frequency range. In all these cases, the value of the superprotonic conductivity was determined from the intersection of the Nyquist plot segments with the Z’ axis at high measurement frequencies. Figure 2d shows the temperature dependence of the proton conductivity measured from Nyquist plots, revealing CDP’s three-order-of magnitude superprotonic jump at T_sp_ = 233 °C.

**Figure 3:**
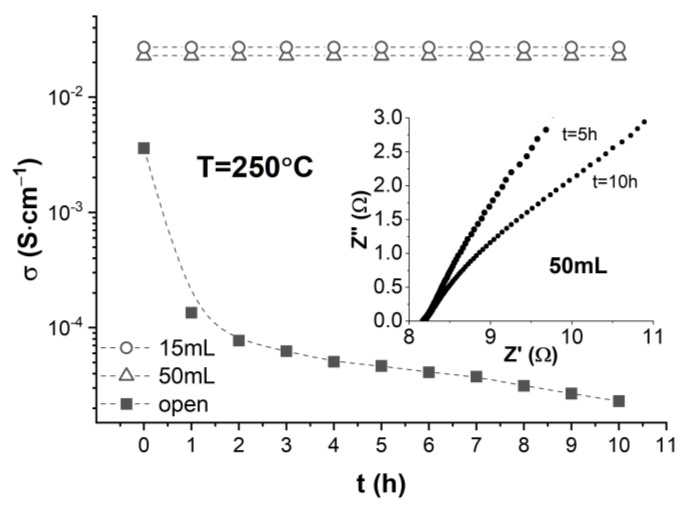
Time dependence of the proton conductivity of CDP measured over a 10 h timespan at T = 250 °C for samples hermetically sealed in 15 mL (circles) and 50 mL (triangles) of dry air, and in an open atmosphere (filled squares). The inset shows that high-frequency segments of Nyquist plots collected at 5 h and 10 h after the onset of the superprotonic conduction in CDP sealed in the 50 mL chamber intersect the real (in-phase) impedance axis at the same value Z′ = 8.16 Ω.

Next, we focused on investigating the stability of CDP’s superprotonic phase and analyzing the conditions that led to the observed behavior. To that end, we set the temperature to 250 °C and repeatedly measured the proton conductivity of the CDP pellet, σ, at 1 h time intervals. The resulting time dependence of the proton conductivity of a CDP pellet hermetically sealed in 50 mL of dry air, σ vs. t, is shown by the triangles in Figure 3 demonstrating that the proton conductivity stays constant at superprotonic values of approximately 2.3 × 10^−2^ S·cm^−1^ for at least 10 h.

This is further illustrated in the inset, which shows that the low frequency ends of the Nyquist plots measured at t = 5 h and t = 10 h after setting the temperature to 250 °C intersect the in-phase impedance axis at exactly the same value where Z’ = 8.16 Ω. The shapes of the Nyquist plots also confirm the superprotonic nature of the conduction within the observed time frame. A similar σ vs. t behavior was found by using a smaller chamber of 15 mL volume (circles) with the only exception that, in this case, the value of the stable proton conductivity was approximately 2.7 × 10^−2^ S·cm^−1^. On the other hand, the σ vs. t dependence measured in an open atmosphere (filled squares) shows that the proton conductivity rapidly decreases by three orders of magnitude, as the superprotonic CDP phase is not chemically stable under these conditions.

The observation of a stable superprotonic CDP phase in pellets of a 1.3 cm diameter and 0.25 cm thickness that were sealed in dry air volumes that differ by a factor of more than three (15 mL and 50 mL) has important implications for clarifying the conditions that are actually responsible for preventing CDP’s dehydration at temperatures above T_sp_. It was recently proposed that sealing a CDP pellet of the size mentioned above in a small volume of dry air (~15 mL) would result in the generation of a saturated water vapor atmosphere upon heating above T_sp_ [23]. It was hypothesized that a hermetically sealed CDP sample *self-generates* the saturated water vapor atmosphere via its partial dehydration according to Equation (1). It was also assumed that dehydration only proceeds along the radial direction of the pellet, and the percentage of the CDP sample that needs to dehydrate to yield a saturated vapor atmosphere (in the 15 mL chamber) was calculated to be 71%. That would leave 29% of unaltered CDP to form a core cylinder that runs all the way from the bottom to the top of the pellet along its main axis. The calculation was based on a water vapor pressure of 5 bar, previously determined through Raman spectroscopy methods [23]. While this is not an unreasonable hypothesis, we find that several of its aspects are not easy to explain. First, it is unlikely that the dehydration (and conversion to Cs_2_H_2_P_2_O_7_) of almost three quarters of the CDP pellet has no effect on the measured proton conductivity σ. As shown by the data (circles) in Figure 3, σ stays constant at ~2.7 × 10^−2^ S·cm^−1^ for 10 h when the sample is hermetically sealed in 15 mL of dry air. On the other hand, the slow and partial dehydration of CDP, which is known to occur in an open atmosphere during the first two hours after the transition [11], leads to a significant drop in σ (shown by the solid squares in Figure 3), although a superprotonic CDP core would still present in the pellet if the hypothesis above were valid. Second, the samples recovered from the chamber upon cooling back to room temperature show no traces of Cs_2_H_2_P_2_O_7_ [21], which can only happen if the water vapor is completely reabsorbed via a reversed reaction where Cs_2_H_2_P_2_O_7_ + H_2_O → 2CsH_2_PO_4_, but we have no knowledge of any experimental evidence that such a reaction actually occurs. Third, and most relevant, our observation of a stable superprotonic CDP phase above T_sp_ in 50 mL of hermetically sealed dry air (triangles in Figure 3) cannot be explained by the above-proposed scenario as the amount of water needed to create a saturated vapor atmosphere in this volume would require the dehydration of ~0.1 mol of CDP, which is twice as much as what the entire pellet contains.

**Figure 4:**
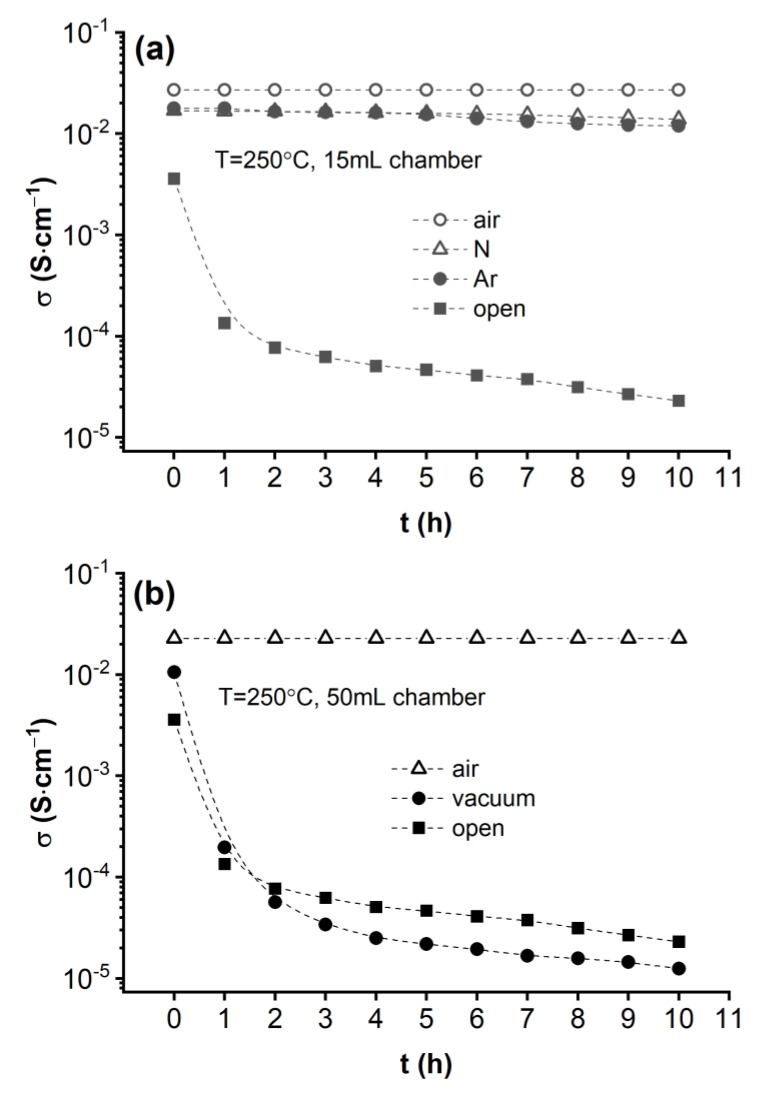
(**a**) Time dependence of the proton conductivity of CDP hermetically sealed in the 15 mL chamber at T = 250 °C in dry air (circles), nitrogen (triangles), argon (filled circles), and in an open atmosphere (filled squares). (**b**) Time dependence of the proton conductivity of CDP hermetically sealed in the 50 mL chamber at T = 250 °C in dry air (triangles) and vacuum (filled circles), as well as outside of the chamber in open air (filled squares).

Clearly, alternative explanations for the conditions and mechanisms that prevent CDP’s dehydration at temperatures above T_sp_ need to be explored. To that end, we measured the time dependence of the proton conductivity on CDP pellets sealed in other environments than dry air at T = 250 °C. Figure 4a shows the σ vs. t|_250°C_ dependence in nitrogen (triangles) and argon (filled circles) atmospheres as compared to their dry air counterpart (circles). The corresponding data collected in an open atmosphere are also shown for comparison (filled squares). These results demonstrate that replacing the dry air with inert gas does not have a pronounced effect on the CDP’s superprotonic phase stability. The proton conductivity changes little in time when compared to the massive decrease observed in an open atmosphere, regardless of the gas used in the chamber. The best stability was achieved in dry air, whereas a slight decrease in σ was observed in nitrogen and argon over a 10 h time period. On the other hand, removing the gas from the chamber to keep the CDP pellet under vacuum has a dramatic effect on its superprotonic phase, which dehydrates even faster than it does in an open atmosphere, as shown in Figure 4b. This shows that the pressure exerted by the gas onto the pellet’s surface plays an important role in preventing CDP’s dehydration above T_sp_. Yet, in all experimental setups used here, the gas pressure in the chamber at 250 °C is about 2 bar (estimated based on an ideal gas pressure increase from RT under a constant volume). This is four orders of magnitude below the value previously used to stabilize superprotonic CDP without hydration, which was 1 GPa [6,11]. Yet, it is important to mention that the choice of 1 GPa in Refs. [6,11] was somewhat arbitrary, and it is possible that CDP stabilization might be achieved at lower pressures.

**Figure 5:**
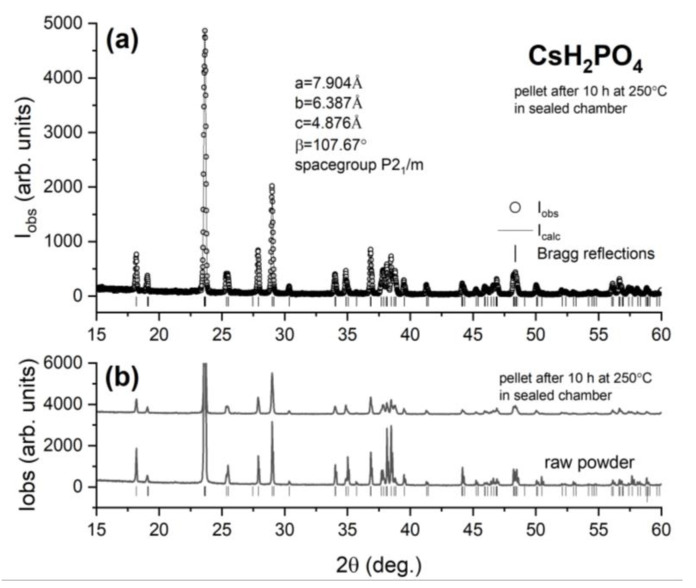
(**a**) Le Bail (full profile) fit to X-ray diffraction data from a CDP pellet after being used in the 50 mL sealed chamber experiment for 10 h at T = 250 °C. The empty symbols show the observed intensity as a function of the detector angle 2θ and the solid line is the fit. The vertical bars mark the positions of the Bragg reflections from CDP’s room temperature. (**b**) Comparison between the X-ray diffraction pattern from the as prepared powder and that from its counterpart collected on the pellet kept for 10 h at T = 250 °C in the 50 mL chamber.

Finally, we investigated the structural and chemical modifications of a CDP pellet kept at 250 °C for 10 h in dry air in a hermetically sealed 50 mL chamber, i.e., under the same conditions where the time-resolved proton conductivity was measured. We collected powder X-ray diffraction data for the pellet recovered from the chamber and compared them with the corresponding data from the raw powder used to press the pellet. These results are presented in Figure 5. The Le Bail fit to the X-ray data (Figure 5a) allows the unit cell parameters to be determined. All observed peaks correspond to Bragg reflections from CDP’s monoclinic modification, whose angular 2θ positions are shown by the vertical bars. No reflections from the dehydration product Cs_2_H_2_P_2_O_7_ were observed. Moreover, a direct comparison between the X-ray diffraction data from the pellet hermetically sealed in dry air for 10 h at 250 °C and its raw CDP powder counterpart (Figure 5b) shows the same peak positions and relative intensities, indicating that the same unaltered room-temperature monoclinic P2_1_/m phase is present in both. Corroborated with the proton conductivity data in Figure 2 and Figure 3, this represents direct evidence that the CDP pellet sealed in the 50 mL chamber undergoes a reversible monoclinic to cubic to monoclinic polymorphic phase transition upon heating above T_sp_ and cooling back to room temperature, with no indication of any chemical decomposition via dehydration. Consequently, our study demonstrates that sealing CDP in small gas volumes represents a new method to stabilize the superprotonic phase without the need to deliver saturated water vapor to the sample or subject it to high pressures in the GPa range.

## 4. Conclusions

We have investigated the chemical stability of CDP’s superprotonic phase at T = 250 °C using X-ray diffraction and AC impedance susceptibility. Data collected for pellets hermetically sealed in small volumes (15 mL and 50 mL) of dry air or inert gas show that CDP’s proton conductivity stays constant at superprotonic values of ~2.5 × 10^−2^ S·cm^−1^ for more than 10 h. We did not find any evidence of a partial dehydration of the sample that would generate high humidity at temperatures above the superprotonic conduction threshold T_sp_. Instead, we observed that completely removing the gas from the chamber leads to a dramatic drop in the proton conductivity of two orders of magnitude in less than two hours. This clearly indicates that the gas pressure plays an important role in preventing the sample’s chemical decomposition. Yet, the gas pressures generated by heating the sealed chambers to T = 250 °C are several orders of magnitude below their high pressure (~1 GPa) counterparts which were previously used to stabilize superprotonic solid acid phases. Consequently, our study demonstrates that hermetically sealing CDP in small gas-filled volumes represents a new method to prevent the chemical decomposition of the superprotonic phases of phosphate solid acids without the need to deliver saturated water vapor to the sample or keep it under high pressure in the GPa range. This finding removes a critical roadblock that, so far, has prevented the large-scale use of these materials as intermediate-temperature fuel cell electrolytes.

## Data Availability

The data presented in this study are available on request from the corresponding author.
